# The unified front: a multidisciplinary approach in the Era of uniportal thoracic surgery

**DOI:** 10.3389/fsurg.2026.1878957

**Published:** 2026-09-24

**Authors:** Aliss Chang, Wei Liu, Joyce Chan, Clarence Chan, Rainbow Lau, Calvin Ng

**Affiliations:** Division of Cardiothoracic Surgery, Department of Surgery, Prince of Wales Hospital, The Chinese University of Hong Kong, New Territories, Hong Kong, Hong Kong SAR, China

**Keywords:** localization, lung cancer, multidiciplinary, multifocal, navigation bronchoscopy, transbronchial ablation, uniportal, video-assisted thoracoscopic surgery

## Abstract

The evolution of thoracic surgery from open thoracotomy to uniportal video-assisted thoracoscopic surgery (U-VATS) represents a paradigm shift toward minimizing surgical trauma. However, the changing landscape of thoracic oncology—characterized by an increase in subsolid nodules, multifocal ground-glass opacities, and locally advanced disease requiring neoadjuvant therapy—demands more than surgical innovation alone. This review explores the critical intersection of U-VATS with multidisciplinary collaboration. We examine how the integration of medical oncology, interventional pulmonology, and radiology enhances the capabilities of the single-port approach. This review will discuss specific combinatorial techniques, including uniportal lung resection following neoadjuvant treatment, navigation bronchoscopic-guided localization strategies, and combining U-VATS with concomitant transbronchial ablation. Finally, we address the logistical and economic challenges inherent in maintaining such complex collaborative frameworks.

## 1 Introduction

Over three decades, thoracic surgery has evolved to minimize surgical trauma while enhancing oncological outcomes. Posterolateral thoracotomy, once the gold standard, has been associated with substantial morbidity, including severe postoperative pain, prolonged hospitalization, and delayed recovery (1–4). Video-assisted thoracoscopic surgery (VATS), introduced in the early 1990s, precipitated a paradigm shift toward minimally invasive approaches, and subsequently, uniportal VATS (U-VATS), initially used for minor procedures (5), was adapted by European surgeons in the early 2010s for major anatomical lung resections (6–8). By employing a single 3–4 cm incision without rib spreading, U-VATS has transitioned from a contentious innovation to an established standard in high-volume centers worldwide.

Concurrently, the thoracic oncology landscape has transformed profoundly. Low-dose computed tomography (LDCT) screening has fundamentally altered disease presentation, with landmark trials demonstrating survival benefits (9–11). Hence, some surgeons may now encounter fewer large central tumors and instead face predominantly early-stage peripheral lesions, often ground-glass opacities (GGO), with a rising incidence of synchronous multifocal cancers creating complex parenchymal preservation challenges (12). Simultaneously, neoadjuvant targeted therapies and immune checkpoint inhibitors are routinely employed in stage II-III non-small cell lung cancer (NSCLC) (13), achieving meaningful tumor downstaging (14–16). However, these therapies may produce dense fibrosis and anatomical distortion, rendering subsequent surgical dissection technically demanding (17, 18).

With this evolving disease spectrum, it is increasingly evident that advances in the uniportal surgical approach alone may be insufficient to optimally address the full complexity of contemporary thoracic oncological cases. To achieve complete resection, maximize functional lung preservation, enhance oncological outcomes, and minimize physiological insult, the U-VATS technique must be embedded in and supported by the specialized expertise of multiple allied medical disciplines in a multidisciplinary fashion.

Accordingly, this descriptive review seeks to synthesize current evidence and accumulated clinical experience regarding the incorporation of U-VATS into a comprehensive multidisciplinary framework. We suggest that the single-port approach achieves its highest clinical and oncological potential when it serves as the central procedural platform of a fully integrated multidisciplinary team (MDT). By examining the interface of U-VATS with medical oncology, interventional pulmonology, and interventional radiology, this review aims to illustrate how collaborative, cross-disciplinary innovations are redefining the scope, safety, and ambition of modern thoracic surgical practice.

## 2 A modern trend in lung cancer surgery—“less is more”

To appreciate U-VATS's role in multidisciplinary care, understanding its historical evolution is essential. Following the first lobectomy for lung cancer in 1910 (19), thoracic surgery has pursued minimizing access trauma while enhancing outcomes and parenchymal preservation. Posterolateral thoracotomy remained the gold standard for over a century (4), offering unrestricted hemithorax access, direct three-dimensional vision, excellent tactile feedback, and optimal maneuverability for complex cases (20). However, this approach caused profound trauma through muscle transection and rib spreading, resulting in severe pain, impaired respiratory mechanics, chronic post-thoracotomy syndrome, and delayed recovery (2, 21, 22). Multiportal VATS in the early 1990s initiated the minimally invasive revolution, eliminating rib spreading and major muscle division, significantly reducing trauma, shortening hospital stays, and improving immune and physical function vs. open surgery (23–27). Recent studies demonstrate survival and functional benefits, such as a parallel study comparing open surgery vs. VATS by Lim et al. showing superior functional scores at five weeks post-VATS (mean difference 4.65, 95% CI 1.69–7.61; *p* = 0.009) (28). A 2026 meta-analysis of 1,185 patients found VATS lobectomy reduced mortality risk by 21% (HR 0.79, 95% CI 0.65–0.96) with similar disease-free survival (29). Comparable outcomes were shown in small cell lung carcinoma (SCLC) resected by VATS (30).

Despite advantages, multiportal VATS requires non-intuitive triangulation due to off-axis camera angles and risks neuralgia across multiple intercostal spaces. U-VATS addresses this through a single 3–4 cm incision, combining VATS's minimal invasiveness with open surgery's intuitive ergonomics (31, 32). The camera and instruments entering the same sagittal plane provide direct, straight-axis viewing, eliminating triangulation (33, 34). Also, single-space access minimizes intercostal nerve injury, reducing postoperative pain, enhancing cosmesis, and facilitating Enhanced Recovery After Surgery (ERAS) pathways (35, 36). While an earlier meta-analysis of 4,142 patients showed comparable operative metrics between approaches (37). Another recent one demonstrated significantly lower pain and opioid consumption for 7 days post-U-VATS among 2,544 patients (38), with equivalent oncological outcomes (39) (Table 1). However, U-VATS presents technical challenges, including concurrent instrument placement, which creates spatial constraints and instrument fencing (40), and therefore entails a steep learning curve (41–43). Therefore, complex cases with adhesions, calcified nodes, or bulky tumors may compromise safety for inexperienced operators. Managing catastrophic hemorrhage requires advanced expertise and a low conversion threshold (44, 45).

**Table 1:** Studies on the benefits of uniportal VATS.

Authors	Study design	Number of patients	Outcomes
Le et al. (31)	Single-center, retrospective study of patients underwent multiportal or uniportal VATS	467 (307 multiportal vs 160 uniportal)	Significant differences in drainage removal time, blood loss, hospitalization duration, postoperative pain No significant differences in disease-free survival and overall survival
Bertolaccini et al. (34)	Single-center posture analysis of surgeons performing either multiportal or uniportal wedge resections	100 (50 multiportal vs 50 uniportal)	The National Aeronautics Space Administration-Task Load indeX (NASA-TLX) for frustration was higher with uniportal VATS (*p* = 0.02) NASA-TLX score for overall workload was higher in triportal VATS (*p* = 0.04) NASA-TLX score for physical demand was higher in triportal VATS (*p* = 0.006)
Harris et al. (35)	Meta-analysis, includes 8 observational studies	1,850 (1,223 multiportal vs 627 uniportal)	Significant reduction in hospital length of stay (*P* < 0.0001), duration of postoperative drainage (*P* = 0.0006), and incidence of overall incidence (*P* = 0.009) in uniportal VATS No significant differences in operative time, perioperative blood loss, or rate of conversion to open thoracotomy
Ng et al. (36)	Single-center retrospective review of patients underwent uniportal VATS	150	Conversion rate = 5.3% No intraoperative or 30-day mortality Overall 2-year mortality rate = 3% At 2-year, disease-free survival rate for stage I NSCLC = 96% At 2 -year, disease-free survival for stage II or greate*r* = 83%
Yan et al. (37)	Meta-analysis, includes 20 studies	4,142 (1,869 uniportal vs 2,173 multiportal)	No significant difference in operative time, blood loss, resected lymph nodes, conversion rate, drainage duration, length of postoperative stay, and pain on the first day
Kanani et al. (38)	Meta-analysis, includes 19 studies	2,544 (1,156 uniportal vs 1,388 multiportal)	Significant reduction in pain at 24 h (*p* < 0.0001) in uniportal group Benefits of pain reduction persisted at 48 h and 7 days Opioid consumption decreased in uniportal group
Ruan et al. (39)	Single-center propensity-match cohort study of patients underwent multiportal or uniportal VATS	106 pairs of matched patients	No difference in intraoperative blood loss, number of retrieved lymph nodes, explored nodal stations, drainage time and volume, and postoperative hospital stay No difference in disease-free survival and overall survival

Concurrent with smaller surgical access, thoracic oncology has shifted toward tissue-sparing resections. While lobectomy was historically standard (46), LDCT screening now detects early-stage peripheral GGOs with indolent behavior. Landmark trials, such as JCOG0802 and CALGB 140503, established anatomical segmentectomy as oncologically equivalent for peripheral ≤2 cm NSCLC (47, 48). This parenchyma-sparing approach is critical for aging populations and multifocal disease (49–51). U-VATS ideally complements this trajectory, as segmentectomies require meticulous distal bronchovascular dissection facilitated by direct, magnified single-port visualization. Modern thoracic surgery now combines least invasive access with highly tailored tissue-preserving operations, demanding accurate nodule localization, margin identification, and, for multifocal disease, hybrid management pathways treating multiple lesions to optimize oncological outcomes and quality of life.

## 3 Challenges in lesion localization—limitations of traditional localization techniques

Accurate lesion localization is essential in surgical and interventional thoracic procedures. As the paradigm of thoracic oncology shifts toward the detection of smaller, early-stage, and frequently subsolid GGOs, intraoperative lesion localization has emerged as a significant technical hurdle, particularly within the constraints of U-VATS. Historically, in open thoracotomy, surgeons relied heavily on manual palpation to identify small or deep-seated pulmonary nodules. However, the transition to minimally invasive techniques severely compromises tactile feedback. In U-VATS, this challenge is magnified. The spatial constraints of a single 3- to 4-centimeter incision preclude the surgeon's fingers from being inserted for direct palpation. Attempting to locate non-visible, non-palpable subsolid lesions using instrument-mediated palpation through a single port can be imprecise and carry risks of parenchymal injury, inadequate oncological margins, or the frustrating necessity of converting to multiportal VATS or open thoracotomy to locate the tumor. Consequently, alternative localization techniques, such as intraoperative lung ultrasound (ILU), image-guided percutaneous localization with hookwire, dye, and/or metallic coils, have been introduced to improve the accuracy of nodule localization before lung resection. While these techniques offer an acceptable localization rate, they also have significant drawbacks. For instance, ILU is a relatively noninvasive technique that can be performed through a single incision and carries a low risk of parenchymal injury. However, it is highly operator-dependent, requires a completely deflated lung to avoid artifacts, and its sensitivity may vary with the depth of the lung lesions (52, 53). Similarly, percutaneous localization gained popularity for its ease of use and high accuracy. Yet the percutaneous approach also has limitations, including the risk of pneumothorax, hemorrhage, patient discomfort, dye diffusion, hookwire dislodgement, and coil migration (54, 55). This disadvantage is especially pronounced when the technique is performed in the radiology suite, and there is idle time between localization and surgery (56–58). Moreover, not all cases are suitable for a percutaneous approach due to anatomical constraints. These drawbacks may potentially render the localization futile upon surgical exploration.

Overcoming the tactile limitations of U-VATS and enhancing the safety and accuracy of localization requires robust, multidisciplinary collaboration, drawing on the expertise of surgeons, thoracic radiologists, and interventional pulmonologists to provide precise, advanced image- or navigation-guided localization.

### 3.1 Preoperative lesion localization—the non-invasive imaging approach

The collaboration with thoracic radiologists has revolutionized preoperative planning and localization, offering essential roadmaps for U-VATS surgeons. One critical contribution is the generation of advanced three-dimensional (3D) radiological reconstructions and segmentation. By transforming standard two-dimensional computed tomography (CT) images into interactive 3D models, radiologists enable surgeons to precisely visualize the spatial relationships among the target nodule, the intricate segmental bronchovascular anatomy, and intersegmental planes. This is indispensable for planning complex, parenchymal-sparing anatomical lung resections via a single port.

Advanced imaging technology allows clear visualization of small, subsolid lung cancers on preoperative imaging, enabling precise preoperative surgical planning to localize lesions by identifying corresponding lung segments and to plan lung-preserving resection with sufficient margins for all lesions. The technique of localization using 3D reconstruction involves identifying fixed anatomical structures relative to the target lesions, such as bronchovascular structures or ribs (Figure 1). Also, intersegmental planes can be identified and used as landmarks for localization or to ensure the resection margin (59). A Chinese group published their prospective results on the use of 3D reconstruction of CT images to localize *ex vivo* lung nodules. Within their cohort, 40 patients with multifocal lung nodules underwent lung resection, and 126 detected lung nodules were resected. 124 (98.4%) were successfully localized by 3D reconstruction of the resected specimen, demonstrating the effectiveness of advanced imaging in localizing multiple lung lesions (60). While this study by Ji et al. demonstrates impressive localization rates, it is important to note that the localization was performed *ex vivo* on resected specimens rather than *in vivo* during surgery. This limits the clinical applicability, as the primary challenge in surgical practice is localizing nodules in the intact lung before resection. Additionally, the study population was limited to 40 patients from a single center in China, which may limit generalizability to other populations and healthcare settings. Thus, this technique is further demonstrated in another prospective *in vivo* cohort of 44 patients with 121 lung nodules who underwent lung resection with preoperative 3D CT reconstruction for localization. Among this group of patients, 117 nodules were successfully localized and resected via U-VATS using wedge resection, segmentectomy, or lobectomy. The remaining 4 nodules were not localized preoperatively because they were larger than 15 mm. No localization-related complications were reported (61) (Table 2). This *in vivo* study provides more clinically relevant evidence. However, methodological concerns should be considered, such as the exclusion of nodules larger than 15 mm, which can introduce potential selection bias. Importantly, this technique requires the operator to have a deep understanding of thoracic anatomy and a clear spatial awareness during the surgery. Additionally, when spatial relationships among anatomical landmarks are lacking or unclear, this technique alone may be inadequate (59, 60). As a result, the learning curve and training requirements for surgeons using this technique should be formally assessed in future studies to facilitate broader implementation of this technology.

**Figure 1:**
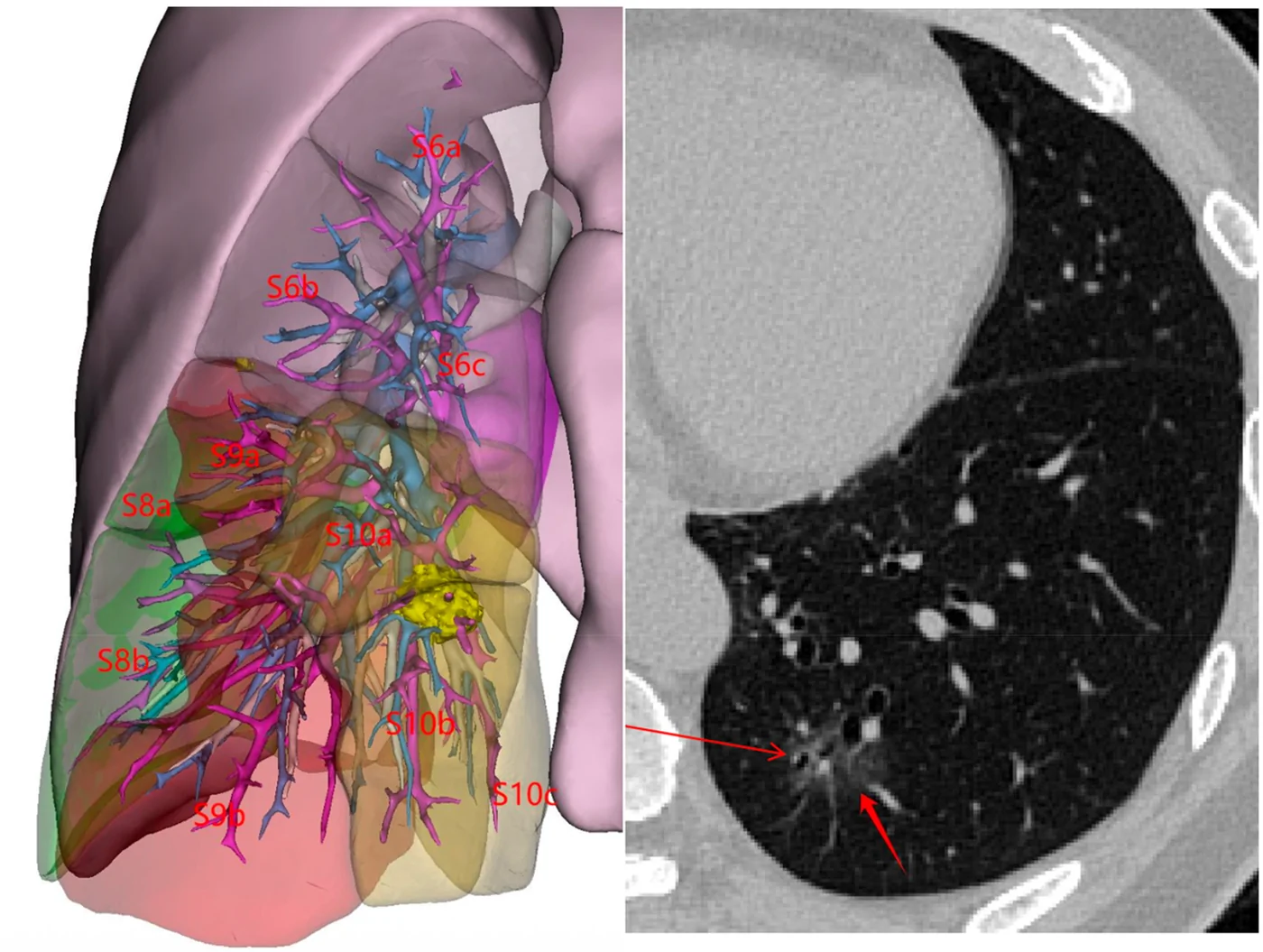
3D segmentation software allows reconstruction of 2D CT images into a manipulable 3D model, showcasing the detailed bronchovascular structures and their spatial relationships with the target lesions (yellow-colored lesions). This can be used to localize the target lesion for U-VATS parenchymal-sparing resection.

**Table 2:** Studies on 3D reconstruction for nodule localization.

Authors	Study design	Number of patients	Outcomes
Ji et al. (58)	Single-center prospective cohort study of patients with multiple lung nodules who underwent lung resection and *ex vivo* nodule localization with 3D CT reconstruction	40 (with a total of 126 nodules)	98.4% of nodules were successfully located in the resected specimens
Zhang et al. (59)	Single-center retrospective cohort of patients with multiple lung nodules who underwent lung resection with preoperative 3D CT reconstruction	44 (with a total of 121 nodules)	All nodules were resected successfully No localization-related complications

In the recent International Delphi consensus report and the European expert consensus, the use of personalized 3D reconstruction and segmentation prior to parenchymal-sparing lung resection is highly recommended (62, 63). In a 2025 retrospective analysis examining the impact of margins on locoregional recurrence and survival, a margin >20 mm was associated with reduced locoregional recurrence and improved survival in sublobar resection (64). Beyond the localization capabilities of 3D reconstruction, a personalized 3D lung model provides a precise map of the bronchial tree, pulmonary vasculature, target lesion location, and intersegmental planes, enabling meticulous resection planning to ensure complete resection of the target lesion and appropriate resection margins, thereby optimizing oncological outcomes. In cases where the adequacy of resection margins may be difficult to determine intraoperatively, preoperative planning with 3D reconstruction may help surgeons decide whether additional localization or marking is required, thereby avoiding intraoperative confusion and preventing a sudden change in the surgical plan.

### 3.2 Minimally invasive transbronchial localization using navigation bronchoscopy (NB)

To circumvent the limitations of percutaneous localization techniques, integrating interventional pulmonology into the perioperative workflow has gained significant traction, particularly through transbronchial localization with NB. Using NB technology, thoracic surgeons or interventional pulmonologists can navigate the complex tracheobronchial tree to the exact site of the peripheral nodule and deploy physical markers or inject liquid dye. The advantages of the transbronchial approach are profound. Because the pleura is not breached from the outside, the risk of procedure-related pneumothorax is greatly reduced (65, 66), and lesions unreachable by percutaneous approach may be accessed via a transbronchial approach. Moreover, when the procedure is performed under general anesthesia immediately before surgery, patient discomfort can be minimized.

Navigation bronchoscopy, including electromagnetic navigation bronchoscopy (ENB) and robotic-assisted bronchoscopy (RAB), represents a significant advancement in tracheobronchial navigation, utilizing technologies such as 3D virtual reconstruction (Figure 2) with real-time electromagnetic guidance or shape sensing (Figure 3), allowing operators to navigate far beyond the optical limits of conventional bronchoscopes, reaching distal airways as deep as the ninth generation with greater stability (67). A propensity-matched study comparing 15 patients undergoing NB-guided localization with 30 undergoing CT-guided localization, which reported similar localization success rates between the two groups (93.3% vs. 90%, *p* = 0.711) and a much lower incidence of pneumothorax in the NB group (6.7% cs 36.7%, *p* = 0.032) (68), positively demonstrating the navigation ability of NB. Additionally, newer robotic systems further enhance this precision by eliminating human-induced instability through articulating robotic arms and remote-control maneuvering (Figure 4). Clinical data, such as the TARGET trials, underscore this technical superiority, reporting navigation success rates beyond 98% using a robotic navigation bronchoscopy system (69). Importantly, the integration of advanced imaging technologies, such as digital tomosynthesis integration into robotic bronchoscopy navigation (TiLT technology, Galaxy System, Noah Medical, California, USA) (Figures 5A,B), addresses the challenge of CT-to-body divergence by providing a continuous, real-time update of the spatial relationship between the navigation tool and the target, thus further enhancing navigation accuracy. The FRONTIER trial is the first-in-human study to investigate the use of the Galaxy System as a navigation tool for lung biopsy, reporting a 100% navigation success rate (70). These demonstrated that the built-in multimodal imaging and robotic arms integrated within these NB systems ensure unparalleled navigational accuracy, making NB an essential tool for precise preoperative localization.

**Figure 2:**
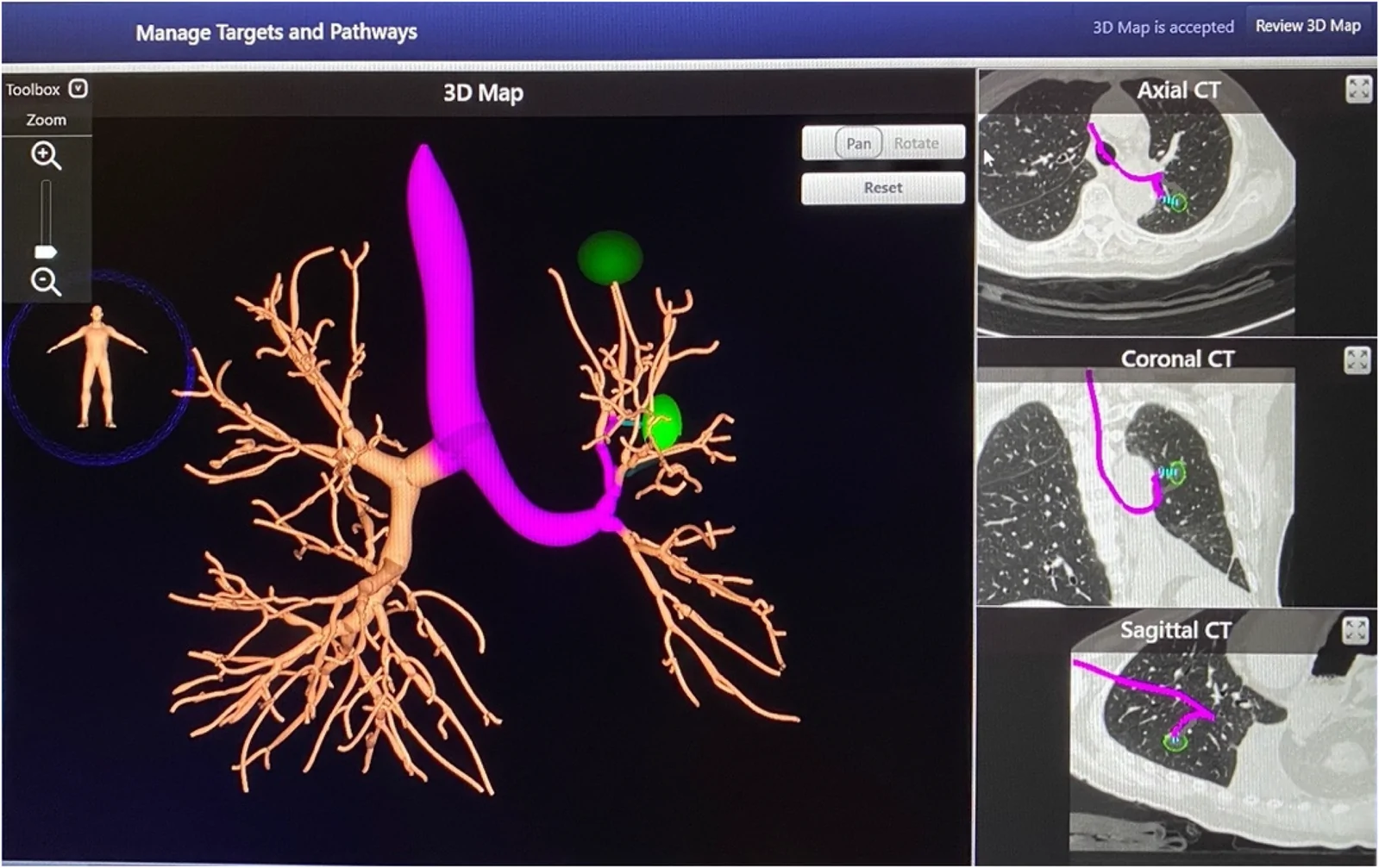
Using planning software from the navigation system, a 3D roadmap of the bronchial tree is created using the pre-procedural CT images. Segmentation of the CT images is done, and the target lesions are virtually projected onto the navigation screen (targets are indicated by the green balls).

**Figure 3:**
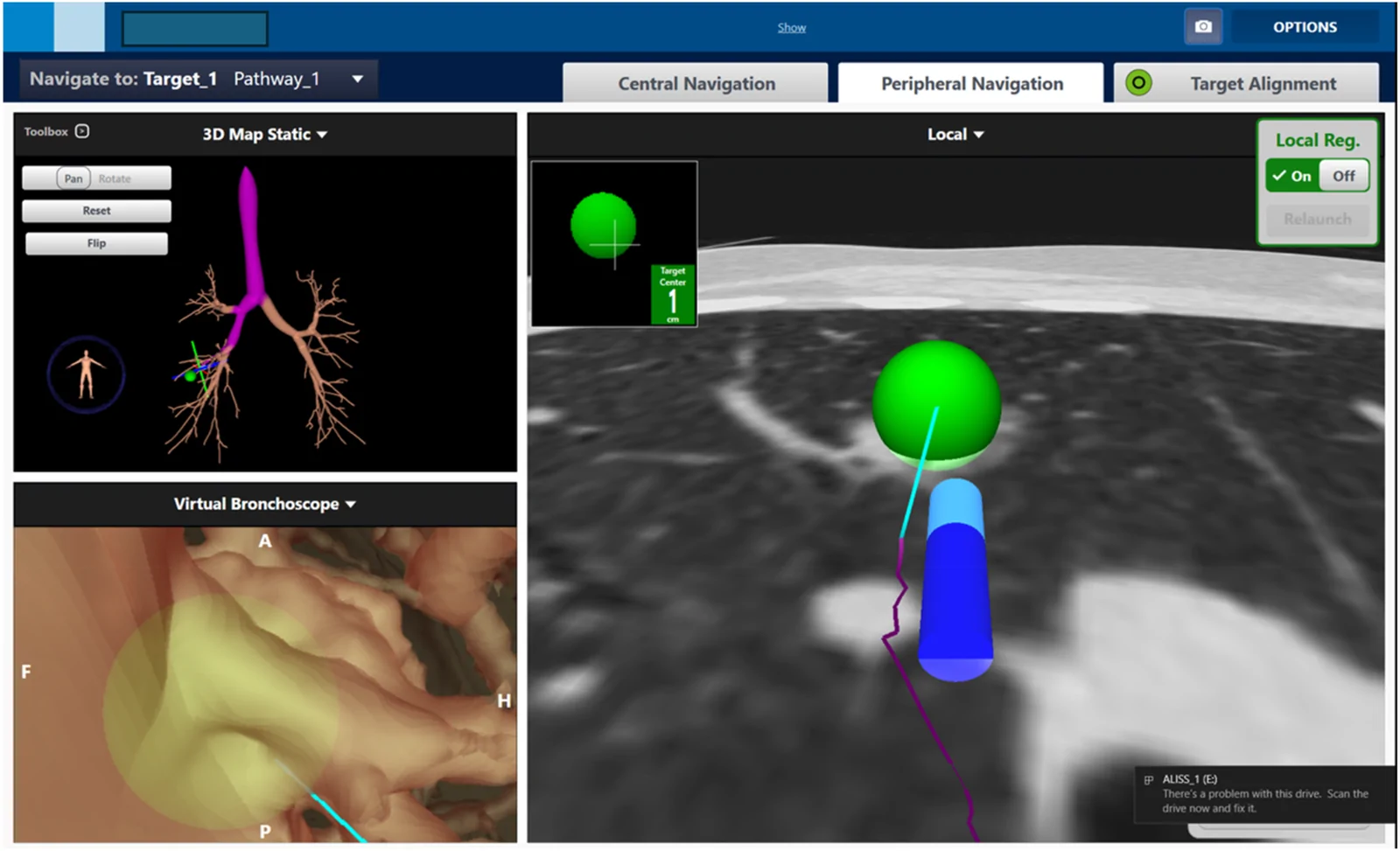
Virtual bronchoscopic view during navigation using electromagnetic navigation, with the target lesion virtually displayed as a green ball. The estimated distance between the tip of the bronchoscope and the target is displayed for ease of operation.

**Figure 4:**
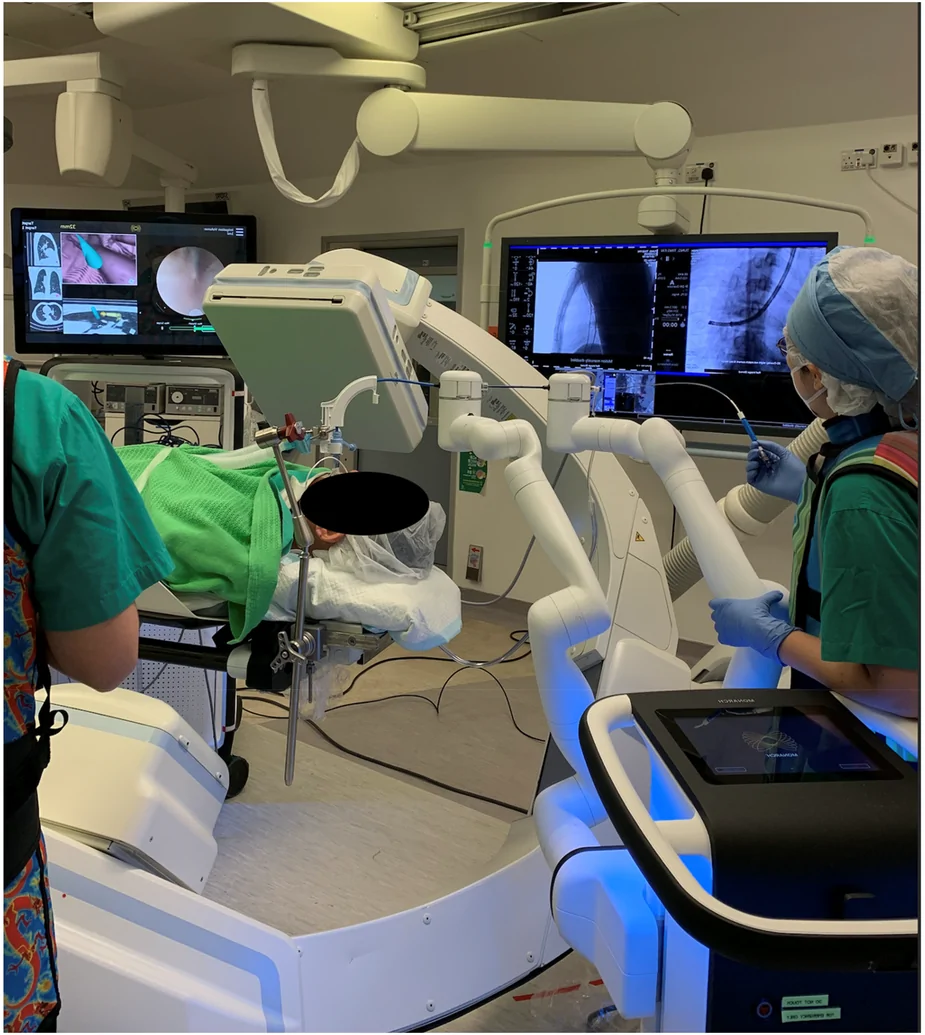
An example of a robotic-assisted bronchoscopy system with a pair of robotic arms controls the articulating robotic bronchoscope, allowing supreme stability. The operator controls the bronchoscope via a remote control, which improves ergonomics during prolonged and complex bronchoscopic procedures.

**Figure 5:**
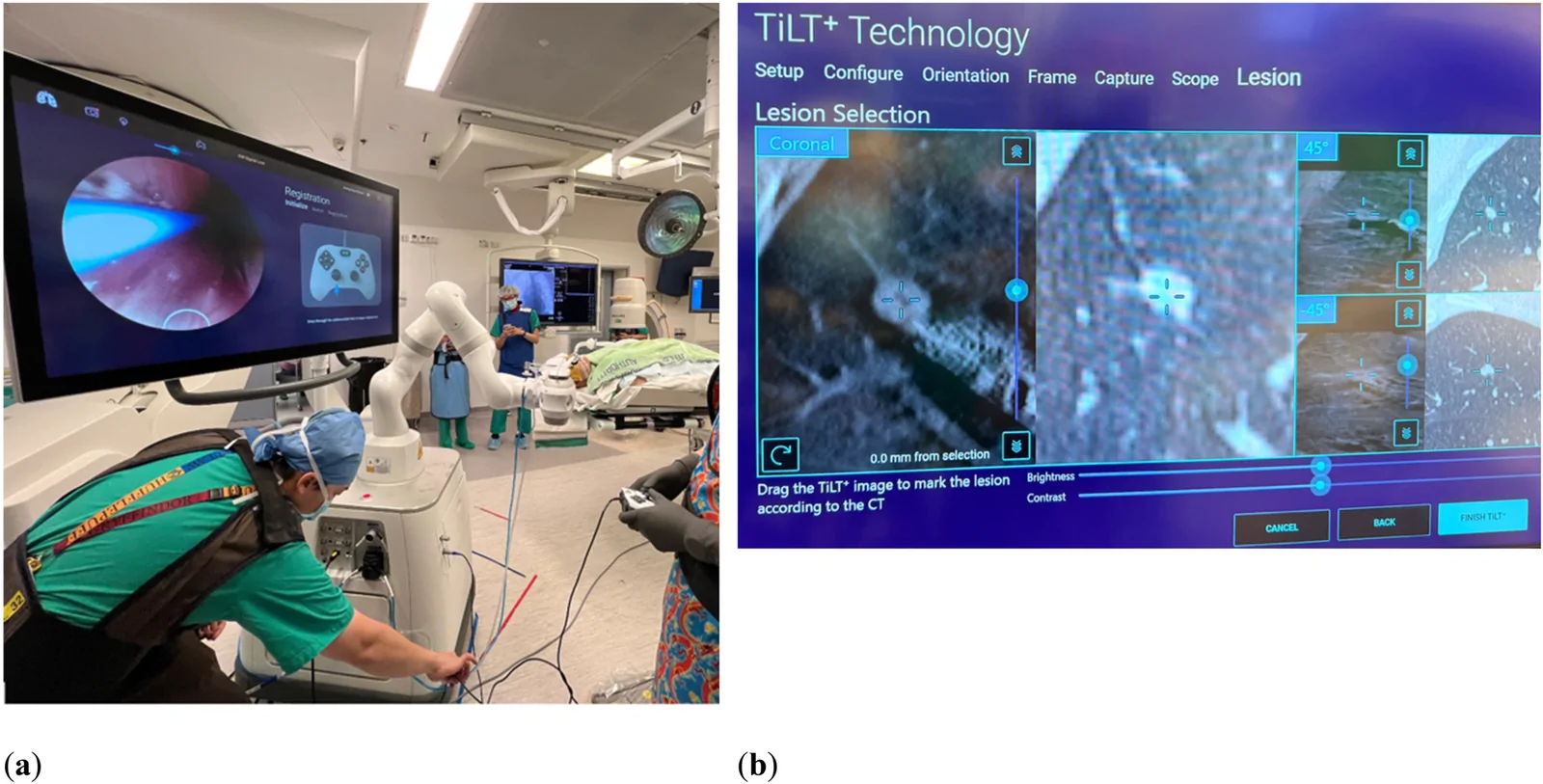
**(a)** A robotic-assisted bronchoscopy system with built-in imaging technologies consists of an articulating robotic arm, which is controlled by a remote control by the operator. **(b)** Tool-in-Lesion Technology (TiLT) uses tomosynthesis to correct for CT-body divergence, updating and realigning the target and instrument location intraoperatively.

Aside from NB's navigational ability, the safety of transbronchial procedures under NB guidance is another notable advantage over the percutaneous approach (65, 66). The transbronchial approach avoids direct injury to the visceral pleura and can substantially reduce the risk of pleural complications, most notably pneumothorax. A retrospective cohort study comparing ENB-guided transbronchial and CT-guided percutaneous localization found equivalent localization success with a lower rate of intraoperative complications in the ENB group (0% vs. 6%) (71). Similarly, Song et al. published a retrospective cohort study that included ENB-guided dyemarking localization in 164 nodules from 134 patients. They reported a localization success rate was 94.5% and a dyemarking success rate of 86.7%. In addition to excellent localization efficacy, no ENB-related complications were noted, suggesting that ENB-guided localization is both effective and safe (72). The safety of NB-guided procedures was further demonstrated in the NAVIGATE study, a prospective, multicenter study involving 1,388 patients who underwent ENB-guided procedures, including biopsy, dye marking, and fiducial marker placement, which was published in 2022 with its 24-month results. Among the 1,329 patients who underwent biopsy, an extraordinary navigation success rate of 94.8% was achieved. Regarding safety, pneumothorax and pulmonary hemorrhage occurred in 4.7% and 2.7% of patients, respectively (73). These results successfully showcase the superior safety profile of NB navigation and localization.

#### 3.2.1 Enhance localization success and convenience with multiple markings

Speaking of the superior navigational accuracy and safety profile, one advantage of using NB-guided preoperative localization is the ability to localize multiple lesions in one go or perform multiple markings to enhance success. A retrospective cohort study was conducted among 203 patients with multiple ipsilateral lung nodules who underwent either ENB-guided or CT-guided dyemark localization. They reported a similar localization success rate between the two groups (97.4% vs. 94.9%, *p* = 0.48). However, the complication rate was significantly lower in the ENB group, with no complications noted, compared to 34.6% pneumothorax and 14.4% hemothorax in the CT group (74). The same group also published a retrospective cohort of 150 patients with multiple bilateral lung nodules who underwent NB- or CT-guided localization prior to VATS. The authors reported comparable localization accuracy with NB and CT guidance. While the localization time using NB localization was much shorter (8 min vs. 25 min, *p* < 0.001), with significantly fewer complications, such as pneumothorax (0% vs. 15.5%, *p* < 0.001) and hemothorax (0% vs. 2.8%, *p* < 0.001) (75), demonstrating the excellent effectiveness and safety of NB localization in multiple lesions. These results can be explained by the avoidance of pleural puncture in ENB-guided procedures, thereby facilitating the localization of multiple lesions in a single procedure. In contrast, CT-guided dyemarking may carry a higher risk of pleural complications and hinder multiple dyemarkings, as a pneumothorax after initial marking may lead to lung collapse, which can then affect the localization of subsequent lesions. Despite these two studies providing impressive safety outcomes and technical feasibility of this technique, these two companion studies from the same research group share similar limitations due to the retrospective nature. The retrospective design and single-center nature limit generalizability, carry potential selection bias, and the lack of randomization introduces potential allocation bias.

Alternatively, multiple markings performed by NB can provide more precise geometric localization, allowing surgeons to visualize the lesion in 3D during surgery. Sato et al. reported their virtual-assisted lung mapping (VAL-MAP) technique in 2013. To perform VAL-MAP, they performed a preoperative 3D CT reconstruction and created a detailed dyemarking plan, which includes multiple sites of dyemarking around the target lesion to ensure complete resection and a personalized resection margin for oncological clearance. Then, virtual bronchoscopic navigation was performed, and dyemarking was performed according to the preoperative plan. After marking, a CT scan with 3D reconstruction was performed to visualize the target lesion and the surrounding markings. This post-marking 3D reconstruction helps surgeons to fine-tune their resection strategies. Using this technique, they have reported their early experience with 30 patients who underwent 95 markings for 37 lung lesions. In the cohort, 92.6% of markings were successful, and all 37 lesions were resected via VATS (100% localization success), with no dyemarking-related complications reported. Despite some failed markings, all lesions can be localized and resected successfully because of the multiple markings, indicating the usefulness of multiple markings for lesion localization (76). Later on, the same group of investigators reported a VAL-MAP 2.0 technique using a deep-margin microcoil marker in conjunction with the original multiple dyemarkings. This technique has been reported to be successful and helpful for achieving accurate sublobar resection, especially for deeply located lesions (77) (Table 3). These two pioneering studies by Sato et al. introduce an innovative approach to preoperative localization. However, several limitations arise from the relatively small cohort and the single-center design at a highly specialized center. Therefore, limiting external validation, generalizability, and the statistical power to detect rare complications. Also, they did not specify the follow-up period for complication assessment, raising questions about whether delayed complications such as delayed pneumothorax or inflammatory reaction occurred. Future research should include multicenter validation studies and standardized complication reporting over longer follow-up periods to establish the true clinical value for VAL-MAP techniques.

**Table 3:** Studies on navigation bronchoscopic localization.

Authors	Study design	Number of patients	Outcomes
Kuo et al. (66)	Single-center 1:2 propensity-matched cohort study of patients who underwent either preoperative ENB-guided or CT-guided percutaneous localization	45 (15 in ENB group vs 30 in CT group)	One failed localization in ENB group vs three in CT group (statistically no significant difference in localization success rate) ENB group has significantly shorter interval between localization to surgery, shorter global procedural time, and lower rate of pneumothorax
Gómez-Hernández et al. (69)	Single-center retrospective cohort study of patients who underwent either preoperative ENB-guided or percutaneous localization	114 nodules (30 in ENB group vs 84 in percutaneous group)	Similar success rates (93.3% in ENB group vs. 91.7% in PM group, *p* = 1). Significantly longer marking time in the ENB group (*p* = 0.007) 6% intraoperative complications in percutaneous group vs 0% in ENB group (*p* = 0.323) Higher radiation dose in ENB group (*p* = 0.002)
Song et al. (70)	Single-center retrospective review of patients who underwent preoperative ENB-guided localization	134 patients with 164 nodules	No ENB-related complications Success rate of dyemarking = 86.7% Success rate of localization = 94.5% Number of dye marking attempts was a significant factor in the localization success rate (*P* = 0.01)
Wang et al. (72)	Single-center retrospective cohort study of patients who underwent either preoperative ENB-guided or CT-guided percutaneous localization	203 patients with multiple nodules in ipsilateral lung (99 in ENB group vs 104 in CT group)	Shorter localization time in ENB group (*p* < 0.001) No related complications occurred in the ENB group (pleural reaction [0% vs. 7.7%, *P* = 0.01], pneumothorax [0% vs. 34.6%, *P* < 0.001], and hemothorax [0% vs. 14.4%, *P* < 0.001] No difference in localization success rate (*p* = 0.48)
Wang et al. (73)	Single-center retrospective cohort study of patients who underwent either preoperative ENB-guided or percutaneous localization	150 patients with multiple nodules in bilateral lungs (79 in ENB group vs 71 in CT group)	Shorter localization time in ENB group (*p* < 0.001) Similar localization success rate Lower pain scores in ENB group (*p* < 0.001) No procedure-related complications in the ENB group (vs 15.5% pneumothorax, 12.7% pulmonary hematoma, and 2.8% hemothorax in CT group, *p* < 0.001)
Sato et al. (74)	Single-center cohort using preoperative VAL-MAP localization	30 (with a total of 37 nodules)	88 out of 95 marking attempts (92.6%) were identified during surgery 100% successful resection rate No procedure-related complications
Sato et al. (75)	Single-center cohort using preoperative VAL-MAP 2.0 localization	6 (with a total of 6 nodules)	All dyemarkings and microcoils were identified during surgery 100% successful resection All macroscopic resection margins were more than 10 mm (17.7 ± 6.3 mm)

#### 3.2.2 Same-session transbronchial localization in the hybrid operating room

NB's astonishing ability to fit into the multidisciplinary management pathway is remarkable. Based on current institutional experience worldwide, the accuracy of NB can be further augmented with imaging adjuncts (78) (Figures 6A,B), especially when performed in the hybrid operating room (HOR). HOR is a fully functional magnetic- and radiation-compatible operating room equipped with various imaging adjuncts, such as cone-beam CT (CBCT), fluoroscopy, and radial EBUS (rEBUS). HOR enables multiple concomitant procedures, offering a one-stop solution for lung cancer management (Figure 7). It enables a “one-stop shop” that streamlines workflows for concomitant procedures, shortens total procedural time, reduces patient discomfort, minimizes delays between procedures, and eliminates risks during patient transfer (79, 80). For instance, Li et al. reported using ENB with rEBUS for transbronchial biopsy of 479 nodules, with a mean size of 20.9 mm. They have achieved a 98.5% visibility rate (81), indicating that imaging adjuncts are useful for further enhancing the accuracy of NB navigation.

**Figure 6:**
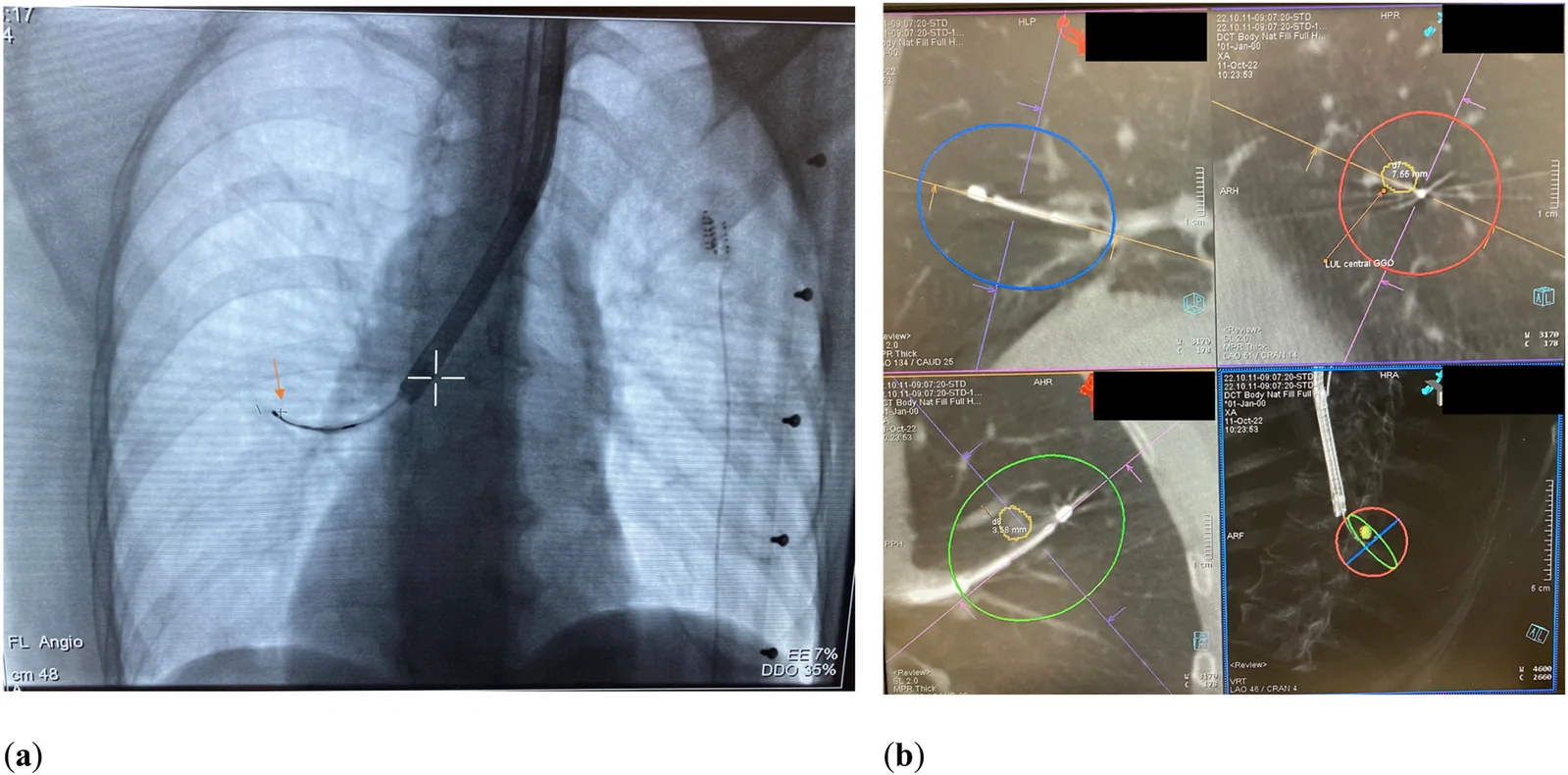
**(a)** Real-time intraoperative fluoroscopy confirmed the target location and the ablation catheter adjacent to the target lesion (indicated by the tiny white cross, pointed by an orange arrow). **(b)** Intraoperative CBCT images confirm the optimal position of ablation catheter placement. The colored lines indicate the predicted ablation zone, which adequately covered the target lesion (yellow lesion).

**Figure 7:**
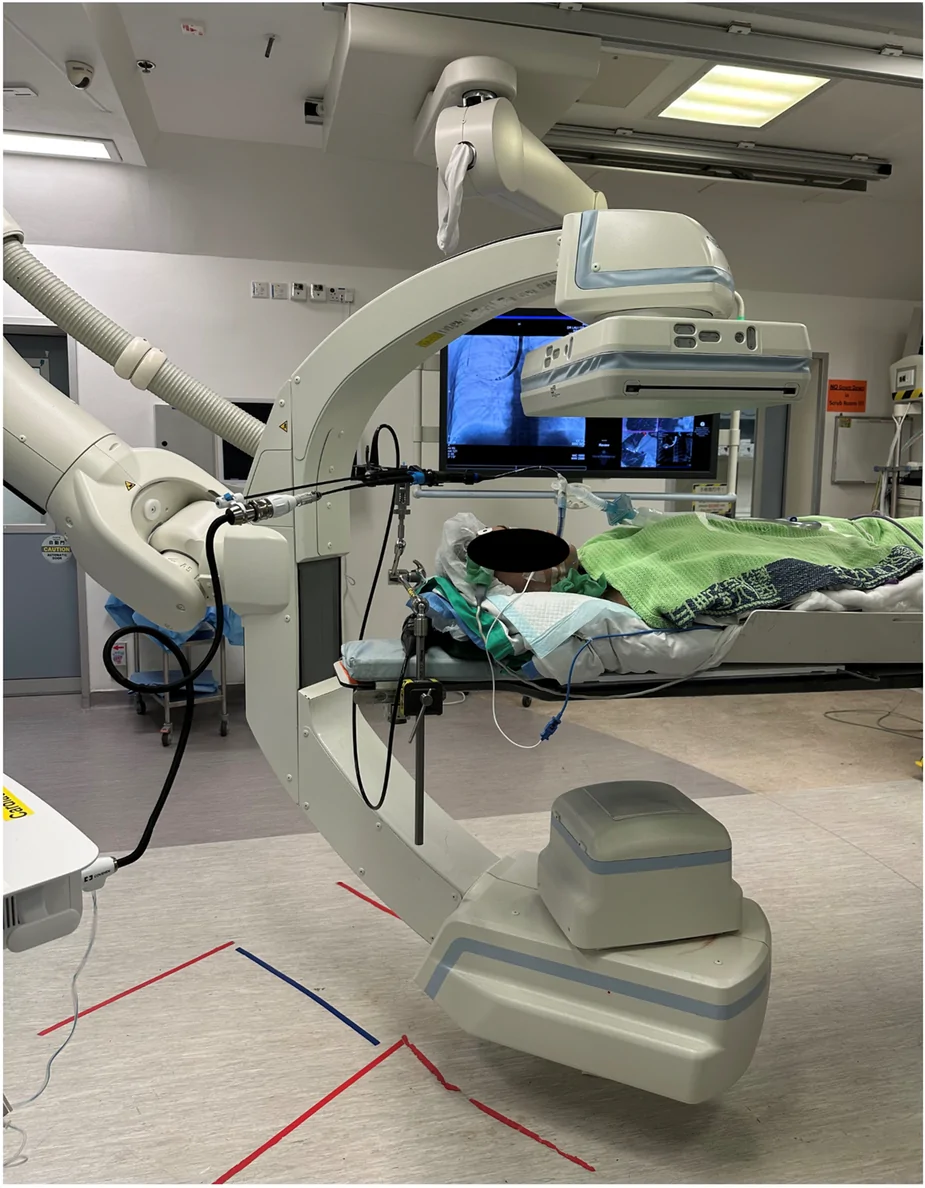
Hybrid operating room set-up for performing ENB MW ablation using CBCT and fluoroscopy as the imaging adjuncts.

Various localization techniques can be used to facilitate precise lung resection surgery (79, 82). The instrument channel in NB permits the passage of an aspirating needle, which can be used to inject liquid dyes, contrast agents, or radiotracers. Likewise, physical markers such as gold fiducial, platinum microcoils, or radiofrequency identification markers can be placed via a needle introducer through the instrument channel (83, 84). Authors have reported their experience with ENB-guided triple dye plus gold fiducial localization prior to lung resection in the HOR. The triple dye concoction is made by mixing Indocyanine Green (ICG), Methylene blue (MB), and Iohexol imaging contrast. ENB navigation was performed in the HOR, and the triple dye was injected near the target lesion through a fine-needle aspiration needle. Afterward, the extended working channel was retracted slightly to the desired deep margin of the target lesion, where a catheter containing the gold fiducial marker was directed through, and the marker was deployed at the deep margin. Throughout the navigation and localization process, CBCT and fluoroscopy overlays were used to confirm the “tool-in-lesion” position and to ensure the dye and marker location (Figure 8). Without delay, the patient can be repositioned in the HOR and proceed directly to the lung resection. During VATS, the naked eye can visualize the target lesion due to the presence of MB. It can also be visualized with a near-infrared camera and x-ray fluoroscopy due to the presence of the ICG and Iohexol contrast agents. On-table fluoroscopy can confirm the inclusion of the gold fiducial marker during stapling, thus securing the deep margin (85, 86). This successfully illustrates the application of concomitant NB-guided localization techniques in the HOR and its ability to create a highly efficient workflow for lung surgery, particularly facilitating MIS with uniportal surgery. An analysis of ENB ICG localization prior to non-intubated U-VATS, reported by Wang et al., included 243 patients with 251 lesions. The navigation success rate was 98.8%, with no localization-related complications and no major postoperative complications (87). Another retrospective study in a similar setting using ENB MB localization, including 57 patients with multifocal lung lesions, reported a localization accuracy of 94% and no localization-associated complications (88). These cohorts confirmed the preliminary efficacy and safety of NB-guided localization in facilitating precise, minimally invasive lung resection (Table 4). To further validate the clinical evidence, a prospective, randomized study is expected to provide comparative data on NB-guided vs. image-guided localization (89). Speaking of shortened procedural time, Kuo et al. performed a series of ENB and CT-guided localizations before proceeding to VATS lung resection. According to their analysis, both methods achieved a high localization accuracy of over 90%. Unsurprisingly, the total procedural time was significantly shorter in the ENB group than in the CT-guided group (68). A shorter idle time between localization and surgery may prevent adverse events, such as dye diffusion, and therefore improve localization accuracy. While the concept of concomitant HOR procedures appears advantageous, the cited studies lack rigorous prospective comparative designs and cost-effectiveness analyses. The critical gaps remain: the absence of randomized controlled trials comparing NB-guided localization in HOR vs. conventional sequential approaches, the lack of independent comparable validation, and the inadequate assessment of the substantial infrastructure costs and training requirements for HOR implementation, which pose major challenges to generalizability across different healthcare settings.

**Figure 8:**
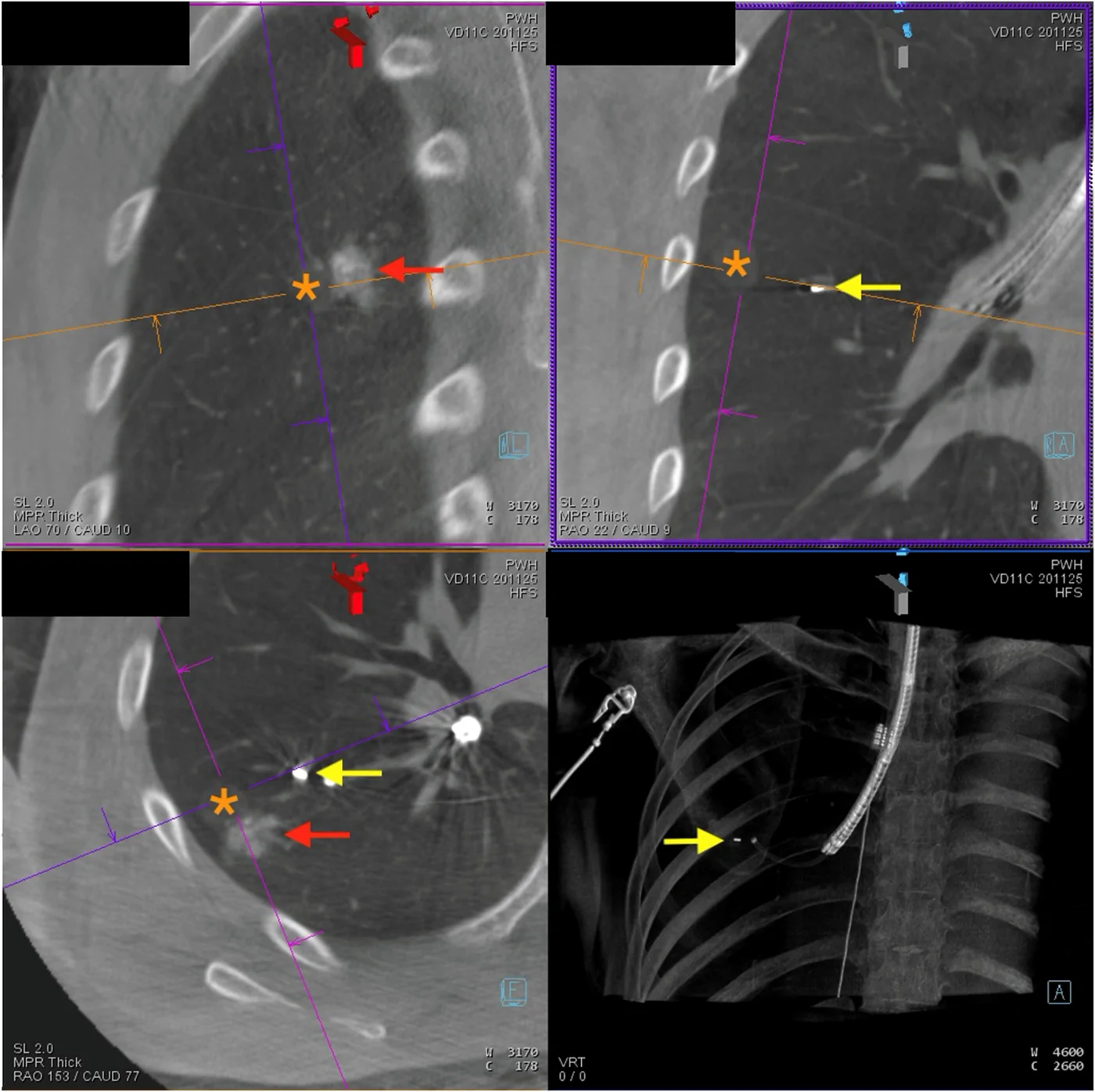
Before lung resection, triple dye plus fiducial marker localization was performed under CBCT and ENB guidance in HOR. Successful marking is confirmed on CBCT. (orange star = target ground-glass lesion, red arrow = triple dye, yellow arrow = fiducial marker).

**Table 4:** Studies on NB-guided localization in HOR.

Authors	Study design	Number of patients	Outcomes
Wang et al. (85)	Single-center retrospective study with patients who underwent ENB dyemarking with same-session U-VATS in the HOR	243 (with a total of 251 nodules)	98.8% successful navigation 100% successful resection for all nodules No serious complications Mean postoperative hospital stay = 1.80 ± 0.83 days
Zeng et al. (86)	Single-center retrospective study with patients who underwent same-session ENB dyemarking with VATS	57 (with a total of 150 lesions)	Overall localization accuracy = 94% (assessed by measuring the marker-to- lesion distance) No localization related complications

For the U-VATS surgeon, visualizing the MB dye with the naked eye, the ICG fluorescence under near-infrared thoracoscopy, and/or the metallic marker under fluoroscopy provides precise, real-time guidance for nodule identification, stapler placement, and margin planning. As parenchymal-sparing lung resection becomes the trend, personalized margin planning is crucial to ensure oncological outcomes (64). NB-guided localization techniques, especially when performed in the HOR, will allow clear visualization of the resection margin and thus ensure margin adequacy. Moreover, these localization methods perfectly compensate for the lack of tactile feedback in U-VATS. Despite these advantages, NB-guided transbronchial localization is not without its drawbacks. The primary cons are the learning curve and cost of NB. These procedures require highly specialized, expensive navigation equipment and access to a HOR, which may not be available in every institute. Furthermore, a skilled surgeon or pulmonologist experienced in interventional procedures is required for effective localization, and a learning curve has been observed in previous studies (90–92). Ultimately, however, by effectively neutralizing the localizing challenges inherent to single-port access, both advanced radiological and transbronchial multidisciplinary approaches empower the U-VATS surgeon to confidently perform highly precise, tissue-sparing resections for early-stage, multifocal lung cancers.

Despite the transformative potential of combining NB and HOR with U-VATS to achieve precise localization and parenchyma-sparing resections, several notable limitations must be acknowledged. From an institutional perspective, the installation and maintenance of an HOR requires immense capital investment, which may be prohibitive for smaller or resource-limited healthcare centers. Logistically, integrating intraoperative CBCT and navigation systems may increase procedural setup and operating room turnover times, potentially reducing overall surgical volume. Furthermore, from a technical perspective, the physical bulk of the advanced imaging equipment (such as a floor-mounted CBCT) may crowd the operating space during bronchoscopy and potentially impede the surgeon's ergonomic positioning, necessitating careful planning of the operating room layout. Lastly, transbronchial localization using NB is fundamentally challenged by “CT-to-body divergence”, a phenomenon in which physiological changes, such as positive-pressure ventilation or atelectasis, cause the actual intraoperative position of the peripheral nodule to deviate significantly from the preoperative planning scan (93). Although the use of advanced imaging in the HOR can reduce the risk of this divergence, clinicians will need to balance the logistical, technical, and economic factors outlined above when using these technologies.

## 4 Challenges in multifocal lung cancer—the lung-preserving approach

There is a contemporary paradigm shift in lung cancer from solitary solid tumors to multifocal subsolid tumors observed globally, and many of them carry malignant potential (94). Thus, multidisciplinary and comprehensive treatment of all potentially malignant lesions should be considered to improve overall survival, avoid repeated sessions of general anesthesia, and lessen the patient's stress and discomfort. However, in patients with multifocal lung cancer detected on LDCT screening or as incidental findings, lesions are often small and subsolid, making treatment of multifocal lung cancer particularly challenging. Theoretically and technically, complete resection of multiple lesions using MIS alone is often possible, but it may sacrifice a significant amount of lung parenchyma for relatively small lesions. Additionally, in an aging population, a significant portion of patients cannot tolerate extensive lung resection due to comorbidities or inadequate pulmonary reserve (95). In selected patients, usually after MDT discussion, concomitant lung resection by MIS with same-session local therapy using NB guidance is possible. In addition to combining ENB-guided localization with U-VATS, other therapeutic approaches can be performed with the assistance of the ENB and the HOR. NB-guided local ablation is an excellent contributor to this hybrid treatment pathway due to its ability to be performed concomitantly to multiple lung lesions (96); it can be performed with same-session lung resection surgery (97–99); and it can be performed in local recurrence or patients with a history of lung resection (99).

### 4.1 Hybrid treatment modalities—concomitant U-VATS plus NB-guided ablation

NB-guided ablative therapy has been advocated over the last decade for its ease of use and treatment efficacy. Among available thermal energy sources, microwave (MW) is the most well-known and widely used in NB-guided ablation. Like localization, NB-guided ablation has a lower risk profile than percutaneous ablation while offering comparable local disease control, owing to the avoidance of pleural puncture and high navigation accuracy (100). The NAVABLATE study, published in 2024, demonstrated outstanding technical success, treatment efficacy, and risk profile. This perspective, 2-center, single-arm study included 30 nodules with a mean nodule size of 12.5 mm, treated with ENB MW ablation. The technical success rate was 100%, and one-month post-procedural imaging showed 100% treatment efficacy. There were 4 cases of complications (13.3%), which included pleuritic chest pain, post-ablation syndrome, hemoptysis, and pleural effusion. No pneumothorax or hemothorax was reported (101). These plausible results were replicated in other studies, including the initial experience of ENB MW ablation from the US and the Asia-Pacific. Pritchett et al. in the US conducted a prospective, multicenter study with 10 patients with 11 tumors treated with ENB MW ablation. The median tumor size was 13 × 14 mm. Both technical success rate and treatment efficacy were achieved in all patients. At the 12-month follow-up, no recurrence was detected, and no significant complication was reported. There were two cases of adverse events reported, including one chronic obstructive pulmonary disease exacerbation and one case of unexpected death on post-procedural day 15 in a patient with chronic lymphocytic leukemia and peripheral vascular disease on Aspirin (102). In the Asia-Pacific region, Chan et al. reported their single-center retrospective cohort of 30 nodules treated with ENB microwave ablation. The reported technical success rate was 100%, and no disease progression was detected at the 12-month follow-up. The rates of pneumothorax and post-ablation reaction were 6.67% and 6.67%, respectively (103). The 5-year results of this retrospective cohort were recently updated and presented. In 5 years, a total of 225 lung nodules were treated by ENB MW ablation. The technical success rate remains 100%, with a low-risk profile, including 3.6% pneumothorax, 2.2% post-ablation reaction, 2.7% self-limiting hemoptysis, and 1.8% pleural effusion (104). These results confirmed the feasibility, mid-term efficacy, and safety of NB-guided MW ablation. Alternatives to MW, such as radiofrequency (RF) (105), cryoablation (CA) (106), vapor thermal ablation (107), and others, are under investigation.

To perform NB ablation as a hybrid procedure with U-VATS, the smaller, deeper lesion can be ablated transbronchially, and the larger, peripheral lesion may benefit more from surgical resection. The rationale behind this hybrid approach is to achieve disease control across all lesions and minimize lung volume loss, particularly when treating a small, deep lesion, for which even a sublobar resection would result in a sizable loss of lung volume (108, 109) (Figures 9A–C). In a retrospective cohort combining ENB-guided MW ablation with lung surgery, 11 cases of concomitant MIS lung resection with ENB MW ablation and 92 cases of post-lung resection ENB MW ablation were performed, treating a total of 142 lung nodules. The overall complication rate was 22%, with most being minor complications (74%). The procedural success rate, including both the technical success of MW ablation and successful lung resection with margins, was 100% in all cases (99). These initial results show that a hybrid approach for multiple lesions is feasible. Another cohort reported 11 cases of ENB MW ablation combined with either ipsilateral or contralateral U-VATS lung resection. In total, 37 lung lesions were treated, including ENB MW ablation for 21 lesions. The technical success rate of ablation was 100%. Only one patient had postoperative pneumothorax and subcutaneous emphysema, which required symptomatic treatment. No other significant complications were reported (110). This demonstrates the feasibility of concomitant procedures with NB-guided MW ablation in the HOR and that their peri-procedural safety outcomes are promising (Table 5). Nonetheless, the long-term clinical outcomes and disease control of this hybrid approach will require further validation through more extensive trials, as the current evidence supporting NB-guided MW ablation in the hybrid management pathway is hampered by methodological limitations that preclude definitive conclusions about its oncological benefits. The cited studies are predominantly small, single-center or dual-center cohorts with limited sample sizes, lacking randomized controlled trial designs or active comparison groups to establish superiority over established treatments such as surgical resection or SBRT. Additionally, these cohorts may include highly selected patient populations or institutional expertise that may not translate to broader practice settings. Future research should prioritize multicenter randomized controlled trials with adequate follow-up to validate oncological and functional outcomes. Particularly when considering the benefits of preserving lung function and enhancing postoperative quality of life, caution is warranted in interpreting the current literature, as most studies lack the granularity to draw definitive conclusions about these advantages.

**Figure 9:**
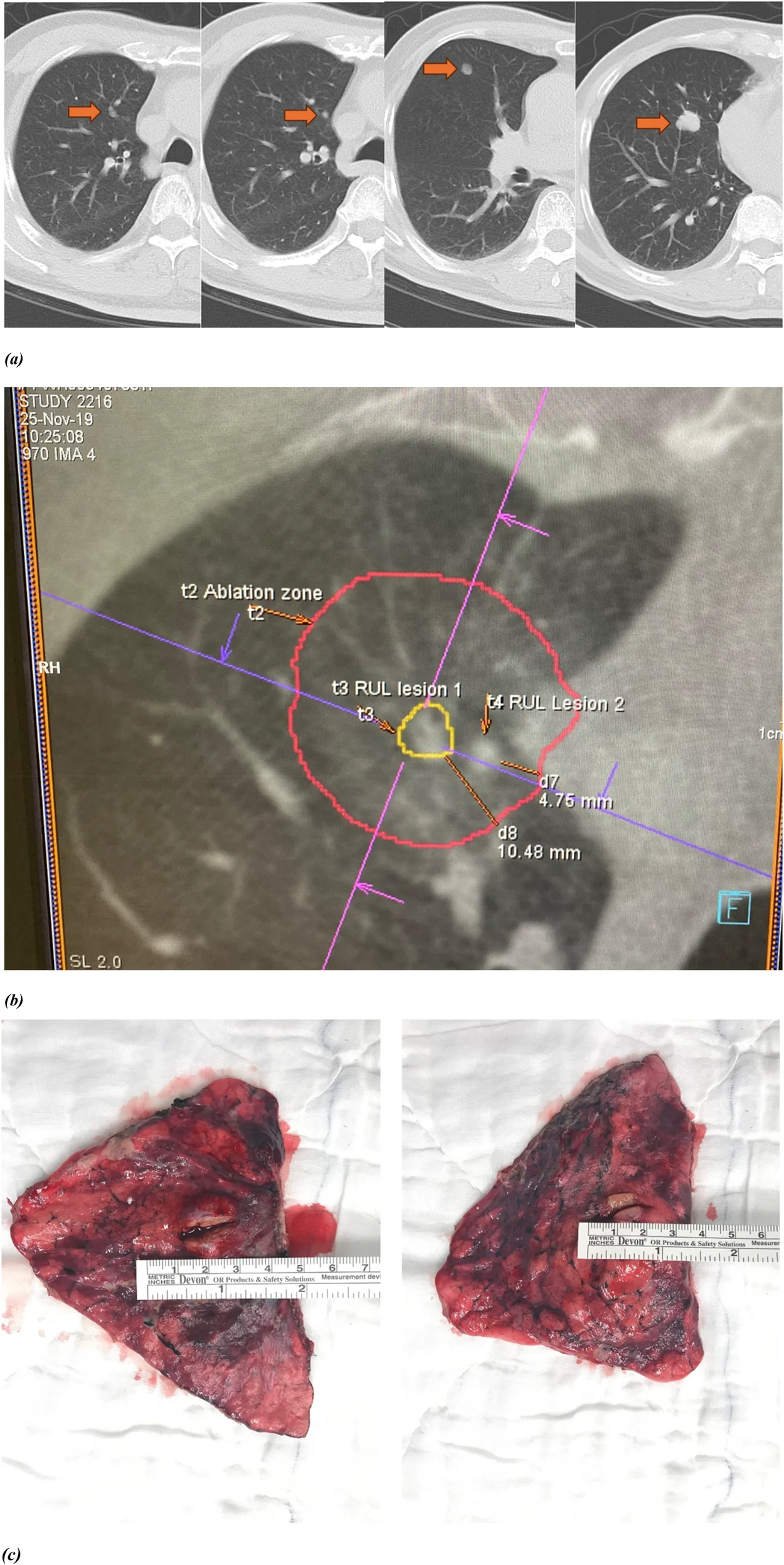
**(a)** In a patient with multiple lung metastases from recurrent hepatocellular carcinoma, hybrid ENB MW ablation was performed to smaller right upper lobe tumors and same-session U-VATS wedge resection was performed for two larger right middle lobe tumors. **(b)** Post-ablation CBCT showed adequate ablation zone covering both right upper lobe tumors. **(c)** Resected right middle lobe specimen showed satisfactory margins with intact tumors within.

**Table 5:** Studies on hybrid treatment using VATS with NB-guided ablation.

Authors	Study design	Number of patients	Outcomes
Chang et al. (97)	Single-center retrospective study in patients who underwent transbronchial microwave ablation with either a history of major lung resection or same-session ablation with concomitant VATS	92 patients (11 cases of concomitant VATS with ablation)	For the concomitant procedure cases (*N* = 11), the average procedural time = 226mins (significantly shorter than cases of separate surgery and ablation) In all patients, technical success rate for ablation = 100% (mean minimum margin = 6 mm)
Qu et al. (108)	Single-center retrospective study in patients who underwent same-session ENB-guided ablation with U-VATS	11 patients (with a total of 37 lesions)	Mean postoperative hospital stay = 6.2 ± 2.3 days One case of postoperative pneumothorax and no other procedure-related complications 100% ablation success rate

While the concomitant use of U-VATS resection and NB-guided local ablative therapies offers a pragmatic, parenchyma-sparing strategy for patients with multifocal lung cancer, this hybrid approach carries certain oncological limitations. The primary drawback of local ablation is the inherent lack of comprehensive histopathological evaluation. Unlike surgical resection, ablation destroys the tissue architecture, thereby precluding definitive assessment of tumor histopathology and, critically, regional lymph node involvement. This absence of localized nodal staging for the ablated lesions raises the risk of clinical under-staging. Furthermore, the molecular landscape of synchronous multiple primary lung cancers is highly complex, characterized by profound tumor heterogeneity. Distinct lesions within the same patient frequently harbor entirely different oncogenic driver mutations. Consequently, with a hybrid treatment combining lung resection and local ablation, even when the primary tumor is resected via U-VATS, providing ample tissue for comprehensive histological subtyping and molecular profiling via next-generation sequencing, clinicians cannot reliably extrapolate these biomarker results to the concomitantly ablated lesions. Although one might theoretically biopsy the secondary nodules immediately prior to ablation, intraoperative biopsies of small, subsolid lesions are technically challenging and frequently yield insufficient tissue for extensive molecular testing (111). Moreover, risks of lung biopsy, such as parenchymal hemorrhage or pneumothorax, if they occur, will significantly hinder the feasibility and efficacy of subsequent ablation. In particular, the occurrence of pulmonary hemorrhage after biopsy might affect the intraoperative radiological assessment of lesion characteristics, ablation zone, and margins. Ultimately, while this “resect and ablate” multidisciplinary approach balances disease control with pulmonary reserve, it may inherently compromise the principles of precision medicine by leaving a critical gap in the comprehensive molecular and pathological staging of the patient's multifocal disease.

### 4.2 Hybrid treatment modalities—U-VATS plus stereotactic body radiation therapy (SBRT)

Aside from local ablative therapies, another well-established form of local treatment is stereotactic body radiation therapy (SBRT). In the latest NCCN guideline, SBRT is considered the standard of care for medically inoperable early-stage lung cancer. The performance of SBRT has been demonstrated in landmark studies, such as RTOG 0236 (112) and JCOG 0403 (113). Among the 55 patients who received SBRT for early-stage lung cancer in the RTOG 0236 trial, the estimated 3-year primary tumor control rate was 97.6%, the 3-year local control rate was 90.6%, and the 3-year local-regional control rate was 87.2%. The 3-year disease-free survival and overall survival were 48.3% and 55.8%, respectively. Regarding safety, they reported a rate of treatment-related grade 3 or 4 adverse events of 16.3% (112). These results were echoed in the long-term follow-up of the JCOG 0236 trial, which consisted of both operable and inoperable patients who underwent SBRT for early-stage lung cancer. In this cohort of 64 patients, the 10-year survival was 23.8%. The 10-year progression-free survival and local progression-free survival were 19.1% and 20.9%, respectively. The 10-year local control rate was 85.4% (113). These trials have demonstrated the local disease-control efficacy of SBRT in early-stage lung cancer, and these results have subsequently prompted investigations into its use to treat multifocal lung cancer. Owen et al. reported a prospective cohort of 63 patients with 128 meta-synchronous and synchronous lung nodules treated with SBRT, with 1-year overall survival of 85% and 1-year progression-free survival of 91.9% (114), indicating the feasibility of SBRT for multifocal lesions.

With the trend toward MIS and lung-preserving lung resection for multifocal lung malignancies, a hybrid approach combining MIS lung resection with SBRT has emerged as a compelling multidisciplinary strategy. The primary advantage of this paradigm lies in its synergistic ability to achieve comprehensive local disease control while maximizing parenchymal sparing. Typically, the dominant, most central, or radiologically aggressive lesion is resected via VATS, providing definitive nodal staging and yielding crucial tissue for comprehensive molecular profiling. While the secondary, contralateral, or peripheral lesions are treated with SBRT. This selective surgical approach spares patients the profound morbidity and functional decline associated with multiple or staged lung surgeries. A multicenter, retrospective study reported results for 203 patients with multiple primary lung cancers who had previously undergone lung resection and were assessed for SBRT or reoperation (73 in the SBRT group and 130 in the surgery group). The reported locoregional recurrence rate and cancer-specific survival were similar (3-year locoregional recurrence was 13.1% in the SBRT group vs. 7.4% in the surgery group, *p* = 0.14; 3-year cancer-specific survival was 88.1% in the SBRT group vs. 93.8% in the surgery group, *p* = 0.23). For safety, 2.7% had grade 3 radiation pneumonitis in the SBRT group, while 3.1% had grade 3 complications and four cases of 90-day mortality in the surgery group (115). This retrospective study supported SBRT as an effective treatment option in the multidisciplinary pathway for multifocal lesions.

However, this hybrid strategy carries notable limitations. Echoing the drawbacks of local ablation, SBRT precludes histological confirmation and targeted mutational analysis of the irradiated nodules. This lack of molecular data can complicate the future application of targeted systemic therapies. Furthermore, SBRT introduces the risk of radiation toxicity, which heavily depends on the anatomical location of the targeted lesions (116). While SBRT demonstrates an excellent safety and efficacy profile for small, peripherally located nodules, its clinical utility is potentially restricted when addressing central or ultra-central tumors. Delivering highly ablative radiation doses in close proximity to critical mediastinal structures, such as the proximal tracheobronchial tree, esophagus, and heart, carries a risk of significant radiation toxicities, including advanced pneumonitis and bronchoesophageal fistulation (116, 117). Consequently, these anatomical factors may dictate treatment allocation within the multidisciplinary strategy. Beyond clinical and oncological considerations, implementing a hybrid surgery-and-SBRT paradigm introduces logistical complexities. Unlike intraoperative local ablation, which can be executed concomitantly with resection during a single anesthetic event in the HOR, SBRT requires specialized linear accelerators and complex dosimetric planning. Consequently, the simultaneous delivery of these modalities is physically impossible, mandating that the patient's therapeutic journey be spatially and temporally uncoupled into two distinct treatment phases. Thus, interdepartmental coordination is of paramount importance to allow optimal sequencing and timing of these interventions. Poor coordination or unanticipated prolonged postoperative recovery can result in delays between the two treatment sessions. Such delay may exacerbate patient anxiety and may pose an oncological risk, allowing disease progression or regional spread. Therefore, the ultimate success of combining surgery with SBRT relies heavily on institutional workflow as it does on the technical execution of the treatments themselves. The current clinical evidence supporting this hybrid treatment is predominantly derived from retrospective institutional series and small prospective cohorts. These studies consistently demonstrate that the hybrid approach is well tolerated, with reasonable local control rates and short- to medium-term overall survival. Nonetheless, the formulation of standardized clinical guidelines remains hindered by a lack of large-scale randomized controlled trials, underscoring the need for further robust investigations to determine optimal patient selection criteria and the ideal sequencing of these effective yet distinct therapeutic modalities.

## 5 Challenges in locally advanced lung cancer—expanding surgical indications

While the management of early-stage, peripheral, multifocal lung cancer relies heavily on precise localization techniques and hybrid surgery plus local ablative therapy, the treatment of locally advanced NSCLC presents a vastly different, yet equally formidable, set of challenges. Historically, patients presenting with stage III disease, characterized by large, centrally located tumors, direct invasion into adjacent mediastinal structures, or mediastinal lymph node involvement, were frequently deemed borderline resectable or entirely inoperable. When surgery was attempted upfront, it typically necessitated open thoracotomies, pneumonectomies, or complex reconstructive procedures such as sleeve resection with reconstruction. It carries a high risk of incomplete resections with suboptimal long-term survival rates (118, 119). The sheer anatomical complexity of locally advanced disease has traditionally pushed the limits of surgical feasibility, making MIS approaches such as VATS, let alone U-VATS, seem contraindicated. To overcome these anatomical and oncological barriers, the multidisciplinary integration of medical oncology has fundamentally altered the treatment algorithm through the application of neoadjuvant therapy. The administration of induction chemotherapy, increasingly combined with targeted therapies or immune checkpoint inhibitors (ICIs) before surgical intervention, serves a dual purpose. Systemically, it aims to eradicate occult micrometastatic disease early in the treatment course (120). Locally, neoadjuvant therapy is effective in inducing significant tumor necrosis and shrinkage, thereby downstaging the primary tumor and clearing involved mediastinal lymph nodes, improving oncological clearance after resection (121). This radiographic and pathological response effectively broadens surgical indications, converting previously borderline resectable cases into viable candidates for curative-intent resection.

Several landmark trials studying chemotherapy, targeted therapy, and ICIs in neoadjuvant or perioperative settings have been published in recent years. For example, the CheckMate 816 trial compared neoadjuvant Nivolumab plus platinum-based chemotherapy or platinum-based chemotherapy alone in resectable stage IB to IIIA lung cancer (15), and the KEYNOTE-671 trial compared neoadjuvant Pembrolizumab plus chemotherapy followed by adjuvant Pembrolizumab with neoadjuvant chemotherapy alone in resectable stage II-IIIB NSCLC (16). These studies not only demonstrated survival benefits of incorporating targeted therapy and ICIs but also showed that surgical resection is possible in the majority of patients who underwent neoadjuvant therapy. However, the administration of neoadjuvant immunotherapy and chemotherapy introduces a new, profound surgical challenge: the “frozen hilum”. While neoadjuvant therapy successfully down-stages the tumor, the ensuing tumor regression may be accompanied by an intense localized inflammatory response, desmoplastic reactions, and dense, distinct hilar fibrosis. The normal, predictable anatomical dissection planes are often obliterated, and the delicate pulmonary vessels become encased in fibrotic sheaths and enlarged, calcified lymph nodes. Dissecting these fragile vascular structures may be hazardous and carries a risk of catastrophic intraoperative hemorrhage. Within the landmark trials concerning neoadjuvant or perioperative therapy, investigators have reported surgical complications of any grade in about 40%–70% of patients (14–16). Indicating that although surgery is feasible after neoadjuvant therapy, it may be technically challenging.

### 5.1 Uniportal approach in post-neoadjuvant therapy lung resection

Interestingly, despite these technical difficulties, the MIS approach using U-VATS is feasible and possibly advantageous in the post-neoadjuvant setting when performed by highly experienced surgeons. A retrospective analysis of 29 patients who underwent VATS following neoadjuvant chemotherapy was reported. Among them, 26 patients successfully underwent U-VATS, with two patients requiring conversion to multiportal VATS and one patient requiring conversion to open thoracotomy (10.3% conversion rate). They have reported intraoperative bleeding from tissue adhesions, an indistinct interface between tissue planes, increased vascular fragility, and perivascular lymph node calcification as possible causes of bleeding leading to conversion. Additionally, postoperative complications occurred in five (17.2%) patients, with no operative mortality (122). Similar findings were echoed in another prospective cohort and a case-matched retrospective study. Ismail et al. reported a prospective cohort of 154 patients who underwent U-VATS lung resection after neoadjuvant chemotherapy. They compared them with 136 patients who underwent U-VATS alone, using a retrospective review of records. They reported no difference in perioperative mortality and overall complication rate between the two groups, and there was no conversion to thoracotomy in the U-VATS group. Notably, the rate of prolonged airleak was higher in the neoadjuvant group (22.2% vs. 5.1%), which the authors postulated was probably caused by chemotherapy-induced inflammatory fibrosis (123). A case-matched retrospective cohort of 120 patients (60 underwent surgery after neoadjuvant chemotherapy vs. 60 underwent surgery alone) showed similar rates of postoperative cardiopulmonary complications, hospital length of stay, and readmission between the two groups. However, the post-chemotherapy group has a significantly higher rate of conversion to thoracotomy (13.3% vs. 0%), due to severe bleeding, metastatic lymph node, and severe adhesion (124). Overall, these results showed that U-VATS resection is feasible after neoadjuvant therapy, but technical challenges remain an important concern (Table 6). The primary benefit of the single-port technique lies in the unique geometric ergonomics. Because the U-VATS camera is positioned in the same sagittal plane as the working instruments, it provides a direct, magnified, and straight-line-of-sight view that closely mimics the perspective of an open thoracotomy. This optimal visual axis allows the surgeon to meticulously navigate the tissue planes and dissect the pulmonary artery and vein branches, a task that is often more cumbersome using the off-axis triangulation of multiportal VATS. Furthermore, utilizing the least invasive surgical access is particularly beneficial for this patient population, whose physiological reserves have already been challenged by systemic treatments. By minimizing chest wall trauma, U-VATS may reduce postoperative pain, preserve respiratory mechanics by avoiding chest wall muscle disruption, and accelerate postoperative recovery. This rapid convalescence is highly beneficial, as it minimizes the hospital length of stay and ensures they maintain the functional status necessary to tolerate subsequent adjuvant therapies, thereby optimizing their overall oncological outcome. As expected, applying U-VATS in this complex scenario demands rigorous multidisciplinary coordination, precise timing of surgery relative to the last dose of the neoadjuvant agent, and advanced surgical proficiency, with a low threshold for conversion to thoracotomy should tissue adhesion prove insurmountable.

**Table 6:** Studies on uniportal VATS for post-neoadjuvant lung resection.

Authors	Study design	Number of patients	Outcomes
Yang et al. (120)	Single-center retrospective study in patients who underwent post-neoadjuvant U-VATS	29	3 patients underwent conversion Mean operating time = 160 ± 38.5 min Mean volume of operative blood loss = 167.8 ± 78.4 mL Mean postoperative drainage time = 5.6 ± 1.9 days Mean postoperative hospital stay = 7.7 ± 1.9 days 17.2% of postoperative complications No 30- and 90-day postoperative mortality
Ismail et al. (121)	Single-center retrospective cohort study of patients who underwent post-neoadjuvant U-VATS vs VATS alone	154 (18 post-neoadjuvant vs 136 surgeries alone)	No difference in operative time (*p* = 0.19), 30-day mortality (*p* = 0.605) No conversion in post-neoadjuvant group No intraoperative mortality in both groups Higher incidence of parenchymal fistula leading to prolonged airleak (*p* = 0.013) and re-operation (*p* = 0.007) were found in the post-neoadjuvant group No significant overall survival at 1- and 2-year (94% vs 85% and 89% vs 85%, *p* = 0.324)
Andolfi (122)	Bi-center retrospective propensity-matched study of patients underwent post-neoadjuvant U-VATS and U-VATS alone	120 patients (60 post-neoadjuvant vs 60 surgery alone)	Post-neoadjuvant group has higher conversion rate (13.3% vs 0%, *p* = 0.003) No significant differences in rate of prolonged airleak (13.3% vs 6.7%, *p* = 0.22), chest tube days (4.6 vs 3.6, *p* = 0.2), length of stay (5.1 vs 4.8 days, *p* = 0.69), and readmission rate (5% vs 10%, *p* = 0.29)

More recently, a single-center retrospective study was presented, evaluating 200 patients who underwent surgery after neoadjuvant immunochemotherapy for central lung cancer. Among the 200 patients, 110 patients underwent preoperative 3D vascular mapping plus U-VATS surgery, and 90 patients underwent U-VATS surgery without preoperative mapping. They reported a noteworthy result, showing a significant reduction in intraoperative complications (18.9% vs. 8.2%, *p* = 0.022), fewer severe intraoperative bleeding (HR 0.48, 95% CI 0.28–0.81, *p* = 0.003), shorter hospital stay (4.8 vs. 6.7, *p* < 0.001), and higher 30-days complication-free survival (92.7% vs. 77.8%, *p* = 0.003) among the patients with preoperative 3D mapping (125). This study indicates that an advanced imaging adjunct may help reduce intraoperative complications, particularly during complex lung resection following neoadjuvant therapy. Once again, this illustrates the potential benefits of a multidisciplinary collaboration in a complex thoracic oncological scenario.

## 6 Challenges and practicality in the multidisciplinary approach

While the clinical efficacy and oncological benefits of integrating U-VATS with different medical specialties are increasingly validated, the practical implementation of these multidisciplinary management pathways may encounter substantial structural and economic hurdles.

### 6.1 Cost-effectiveness and resource allocation

Foremost among these challenges are cost-effectiveness and the justifiable allocation of resources. The establishment of comprehensive, multidisciplinary programs requires substantial initial expenditures. The construction of the HOR, the procurement of advanced robotic or electromagnetic navigation platforms, and the continuous use of specialized consumables for NB-guided procedures significantly increase direct procedural costs per case. Some studies have shown that the resulting reductions in postoperative length of stay and complication rates may ultimately offset these initial investments (126, 127). Robust and long-term health economic evaluations and cost-effectiveness analyses remain unverified. Consequently, smaller local healthcare facilities and resource-limited institutions may find the financial burden for adopting these advanced multidisciplinary workflows prohibitive, potentially exacerbating regional disparities in access to cutting-edge thoracic care.

### 6.2 Logistical complexity

The logistical complexity in orchestrating these collaborative interventions cannot be overstated. The successful execution of a hybrid U-VATS procedure relies on the precise synchronization of multiple highly specialized professionals, demanding exceptional administrative agility to coordinate the schedules of interventional pulmonologists, thoracic anesthesiologists, radiologists, and thoracic surgeons. Central to navigating this complexity is the indispensable role of the formal MDT meeting. For complex clinical scenarios, such as multifocal lung cancer requiring surgery with concomitant transbronchial localization or ablation, or borderline resectable NSCLC best managed with neoadjuvant therapy with surgery, routine preoperative case discussions are absolutely paramount. These formalized MDT meetings provide a critical forum for different specialties to engage in shared, evidence-based decision-making. By meticulously reviewing advanced imaging, delineating the exact sequence of perioperative interventions, and establishing clear contingency plans, the MDT ensures highly individualized patient selection and constructs a cohesive treatment package.

### 6.3 Learning curves and expertise

The successful implementation of multidisciplinary U-VATS hybrid management demands proficiencies of multiple complex technologies simultaneously, creating learning curve barriers that may limit widespread adoption, especially in smaller volume centers. For instance, previous literature reported that 50–100 cases are required to achieve technical proficiency and reduced operative times in U-VATS alone (41, 128). Integration of navigation bronchoscopy for localization or ablation as hybrid management with U-VATS will further necessitate competency in endoluminal skills, with studies suggesting 40–50 procedures are required for acceptable ablation outcomes and localization accuracy (91, 92). High-volume centers with dedicated training and mentorship from experienced surgeons may demonstrate superior outcomes. However, many centers may lack such infrastructure, creating a dilemma where novel technologies concentrate in high-volume institutions while community hospitals may struggle with technology adoption. Moreover, the cumulative learning curve across all integrated components may exceed hundreds of cases before consistent outcomes, representing a substantial investment of operative time and institutional resources.

## 7 Future direction—integration of robotic technologies

Looking forward, one of the most transformative future directions for MIS in thoracic oncology is the integration of single-port surgical philosophies with advanced robotic platforms. Over the past decade, robotic-assisted thoracic surgery (RATS) has rapidly expanded as a sophisticated alternative to traditional VATS. Conventional multiportal RATS overcomes the intrinsic ergonomic limitations of standard VATS by providing surgeons with three-dimensional, high-definition binocular vision, tremor filtration, and fully articulating wristed instruments with higher degrees of freedom. These technological enhancements significantly facilitate meticulous dissection and intricate maneuver. Clinically, this translates into better surgical outcomes, including reduced intraoperative blood loss, more extensive lymphadenectomies, shorter chest tube durations, and more (129). However, traditional multiportal RATS inherently necessitates three to four distinct intercostal incisions, thereby sacrificing the minimized chest wall trauma, better cosmesis, and reduced postoperative neuralgia that define the U-VATS approach (130). To reconcile the unmatched ergonomic superiority of robotics with the physiological benefits of single-incision access, the surgical community is now pioneering uniportal RATS (U-RATS). Initial forays into U-RATS attempted to adapt conventional multi-arm robotic platforms into a single incision. While feasible in expert hands (131, 132), funneling multiple bulky robotic arms through a single 3- to 4-centimeter space often results in frustrating external arm collisions, restricted intracorporeal range of motion, and the need for counterintuitive, cross-handed surgical maneuvers.

The true paradigm shift in this domain is the recent development and application of purpose-built, dedicated single-port (SP) robotic surgical systems, for example, the da Vinci SP platform (Intuitive Surgical, California, USA) and the Shurui SP robotic system (Shurui Technology Co Ltd, Beijing, China). Compared with multiportal robotic systems, which achieve triangulation via spatially separated external portals, the dedicated SP system employs a fundamentally different biomechanical approach. It deploys a flexible 3D camera alongside three fully articulating, snake-like instruments through a single specialized cannula. Once inserted into the thoracic cavity, these instruments can diverge and articulate internally, effectively restoring triangulation and providing multidirectional tissue traction without requiring multiple chest wall incisions. This technology eliminates the “chopstick effect” that plagues traditional U-VATS and circumvents the external arm clashing of adapted multi-arm U-RATS, allowing surgeons to perform lung resection efficiently and safely (133, 134).

Crucially, beyond the mechanical advantages, the advent of SP robotic systems holds profound implications for the multidisciplinary treatment paradigms discussed. By coupling the unparalleled precision of robotic dissection with the minimal physiological insult of a single incision, SP RATS has the potential to optimize highly complex hybrid approaches. In the context of post-neoadjuvant therapy, the wristed articulation and tremor filtration may better facilitate the safe dissection of fibrotic hilar structures, potentially reducing intraoperative complications while ensuring the rapid recovery necessary for subsequent systemic treatments. Lee et al. reported a retrospective cohort of 24 patients who underwent lung resection after neoadjuvant therapy, comprising 10 in the SP RATS group and 14 in the U-VATS group. The results showed comparable surgical outcomes between the two groups, including rates of R0 resection, intraoperative conversion, operative time, lymph node yield, chest drainage volume, hospital stay, postoperative complications, and postoperative pain, indicating that SP RATS is feasible and safe in the post-neoadjuvant setting (135). Ultimately, integrating SP RATS into the multidisciplinary framework has substantial potential to further minimize the invasiveness of these hybrid approaches.

As these SP robotic platforms undergo refinements and gain broader regulatory approval for thoracic indications, they are poised to become the new standard-bearer in thoracic surgical oncology, representing the convergence of maximal operative precision and minimal surgical trauma. Nevertheless, while preliminary data from small, single-center cohorts demonstrate reasonable initial safety profiles and promising perioperative outcomes, the clinical evidence base remains in its early stages. To validate its clinical efficacy and cost-effectiveness, large-scale, multicenter prospective trials are essential to accurately compare the long-term oncological efficacy, functional recovery, and health economics of SP robotic systems against established U-VATS and multiportal RATS approaches.

## 8 Limitations

It is important to acknowledge the fundamental limitations of this descriptive review when interpreting its conclusions. First, by its nature as a descriptive rather than a systematic review, the selection and synthesis of the literature are subject to inherent selection bias, as it does not employ the exhaustive, algorithmic search criteria utilized in meta-analyses. More critically, the primary evidence supporting many of these advanced multidisciplinary techniques, such as NB-guided localization, post-neoadjuvant lung resections, and use of HOR, is predominantly derived from retrospective cohort studies, case series, and single-institution experiences. There is currently a distinct lack of large-scale, prospective, randomized controlled trials that directly compare these highly specialized techniques with traditional approaches in standardized settings. Consequently, while the integration of these techniques demonstrates clear clinical feasibility and promising short-term outcomes, the ability to draw definitive, statistically robust conclusions regarding long-term oncological survival and standardized safety profiles remains fundamentally limited. This highlights a critical gap in the current literature and underscores the imperative for future rigorous, multicenter prospective investigations to validate these multidisciplinary paradigms.

## 9 Conclusion

In conclusion, the evolution of thoracic surgery from open thoracotomy to U-VATS represents a profound philosophical shift toward minimizing physiological trauma while maximizing quality of life. As the landscape of thoracic oncology grows increasingly complex, this review suggests that U-VATS may achieve enhanced potential when integrated within a robust multidisciplinary framework, incorporating advanced navigation bronchoscopy-guided transbronchial localization, concomitant ablation or SBRT, and neoadjuvant therapy to better navigate modern lung cancer treatment complexity. However, it is critical to acknowledge that current supporting evidence derives predominantly from retrospective cohort studies and smaller prospective cohorts, limiting definitive conclusions and underscoring the essential need for future large-scale, prospective randomized controlled trials to rigorously evaluate comparative effectiveness, safety, and oncological outcomes. While preliminary data are encouraging, the true clinical value of multidisciplinary U-VATS frameworks remains to be established through higher-quality evidence. Ultimately, the next frontier in thoracic oncology will likely be defined not merely by minimal surgical incision but by seamless multidisciplinary integration of comprehensive perioperative management, ensuring that precision surgery complements precision medicine.
